# Real-World Analysis of the *EGFR* Mutation Test in Tissue and Plasma Samples from Non-Small Cell Lung Cancer

**DOI:** 10.3390/diagnostics11091695

**Published:** 2021-09-17

**Authors:** Hyunwoo Lee, Joungho Han, Yoon-La Choi

**Affiliations:** 1 Samsung Medical Center, Department of Pathology and Translational Genomics, Sungkyunkwan University School of Medicine, Seoul 06351, Korea; hwpatho.lee@samsung.com (H.L.); joungho.han@samsung.com (J.H.); 2 Department of Health Science and Technology, Samsung Advanced Institute for Health Sciences and Technology, Sungkyunkwan University, Seoul 06351, Korea

**Keywords:** lung neoplasms, non-small cell lung cancer, circulating tumor DNA, epidermal growth factor receptor, prognosis

## Abstract

Molecular evaluation of *EGFR* mutation is indispensable in treating non-small cell lung cancer (NSCLC). We compared the results of *EGFR* analysis using tissue DNA (tDNA) and circulating tumor (ctDNA) to evaluate the feasibility of plasma as an effective material for detecting *EGFR* mutation and the reliability of ctDNA analysis in real-world practice settings. We enrolled 554 NSCLC cases who had undergone ctDNA *EGFR* analysis between January 2019 and March 2020. *EGFR* mutations were detected in 240 (57.3%) of the 421 cases with *EGFR* mutations confirmed by tDNA analysis. In multivariate analysis, the size of the largest tumor deposits, disease progression, M stage, the detectable amount of tumor tissue with *EGFR* mutation in distant metastasis, liver metastasis, pleural seeding, and bone metastasis (*p* < 0.05) were identified as independent factors affecting the detection rate of *EGFR* mutations in ctDNA. Survival analysis revealed ctDNA status and M stage (*p* < 0.001) to be independent predictors of overall survival in the multivariate analysis. Our study demonstrates that *EGFR* analysis using ctDNA is a useful clinical tool and can aid in therapeutic decisions in real-world practical settings. However, clinicians should be aware of the possibility of false negatives and confirm *EGFR* analysis using tDNA in certain situations.

## 1. Introduction

Tyrosine kinase inhibitors (TKIs) are one of the most revolutionary classes of therapeutic compounds in medical history. Epidermal growth factor receptor (EGFR) inhibitors are a highly effective treatment [1] for lung cancer, one of the leading causes of death worldwide [2]. Furthermore, the prevalence of *EGFR* mutation in non-small cell cancer (NSCLC) of Asian patients is about 40~65% [3,4], which is much higher than other regions [5]. Therefore, tumor genotyping, including *EGFR* mutation testing, is a particularly important step in predicting sensitivity to targeted therapies in many patients diagnosed with NSCLC, especially in Asia. Nevertheless, acquired resistance cannot be avoided even in patients with excellent therapeutic effect following initial TKI treatment [6,7]. Resistance mutations [8,9], such as the T790M missense mutation in exon 20 of *EGFR*, play an important role in developing resistance to EGFR inhibitors, forcing researchers to focus on compounds that can overcome this resistance in NSCLC [10,11]. However, genotyping of tumors, including *EGFR* analysis, can be limited by the need for tumor tissue samples that are collected via biopsy, which is often contra-indicated in patients with advanced disease.

This means that the development of non-invasive techniques for tumor genotyping has remained at the forefront of cancer research for some time, resulting in the identification of the circulating tumor DNA (ctDNA) assays used in *EGFR* typing and NSCLC evaluations, which rely on the DNA released from circulating tumor cells for analysis [12,13,14,15]. Although ctDNA usually relies on the DNA released from circulating tumor cells within the bloodstream, DNA released from tumor cells undergoing necrosis or apoptosis could also be used. This suggests that apoptotic or necrotic tumor cells from the primary tumor site could also be used for analysis. In addition, ctDNA-based genotyping can be completed at any time during the course of the disease, facilitating real-time detection and monitoring of genotype changes [16]. Several studies [17,18,19,20] have shown that ctDNA status can aid in the prediction of early relapse, allowing clinicians to select suitable candidates for targeted therapy and determine the prognosis and treatment effect of patients during and after treatment. The U.S. Food and Drug Administration (FDA) approved several therapeutic compounds and their companion diagnostic devices—therascreen *EGFR* RGQ PCR Kit, cobas *EGFR* Mutation Test v2, FoundationOne CDx, Oncomine Dx Target Test, Guardant360^®^ CDx, FoundationOne^®^ Liquid CDx—to identify patients who would benefit from treatment [21,22]. In South Korea, the National Health Insurance Service has covered two *EGFR* analysis tests in NSCLC patients—cobas *EGFR* Mutation Test v2, and PANAMutyper^TM^ R *EGFR* [23]. Recently, droplet digital PCR-based *EGFR* test using tDNA—GenesWell dd*EGFR* Mutation Test—has also been included in the health insurance coverage list.

The two methods of using tissue DNA (tDNA) or ctDNA have their respective limitations, so it is important to identify the advantages and disadvantages in real-world medical sites and use them to select patients. In comparison with tDNA profiling, a test using ctDNA is relatively new to clinical sites. The sensitivity for some genetic alterations in plasma or serum samples can be very diverse; thus, some mutations may not be detected when the tumor burden is very low. In addition, the size of the DNA may be reduced to less than 180 bp due to fragmentation, making detection more difficult [24]. The sensitivity of ctDNA analyses may vary with efficiencies between 43% and 93% reported in the literature [18,19,23,25], and these assays have a relatively high probability of returning false negatives. Despite its risk of false negatives, ctDNA analysis has been widely used because it is relatively free from adverse events, possibly caused by invasive procedure and can provide sufficient useful information in clinical situations. It is a good alternative approach for patients with difficulty in obtaining tissue, but there are limitations of the test. It is becoming increasingly important to be clearly aware of the deficiency in that patients should not be deprived of treatment opportunities.

In this study, we planned to investigate how this ctDNA analysis has been used in real-world clinical settings and whether the results are clinically useful. Since it is well known that sensitivity of ctDNA analysis could be affected by a number of factors, we would like to find out whether the same problems occur in real-world clinical settings and to evaluate what factors need to be considered to improve sensitivity.

## 2. Materials and Methods

### 2.1. Case Selection

We collected all of the ctDNA *EGFR* results completed at the Samsung Medical Center between January 2019 and March 2020. This study was approved by the Institutional Review Board (IRB) of the Samsung Medical Center (IRB No. 2020-05-129). Informed written consent from patients was waived by the IRB of Samsung Medical Center because of the retrospective study design.

### 2.2. Clinicopathological Review

The results of computed tomography (CT), magnetic resonance imaging (MRI), and positron emission tomography (PET) CT were all collected, and the TNM stage was determined using both imaging and pathological reviews. The size of the tumor was determined based on the largest diameter within the primary or largest tumor deposit.

We obtained the hematoxylin and eosin-stained slides from the formalin-fixed paraffin-embedded (FFPE) tissues of all patients who underwent surgery and used these to complete the comprehensive pathological review. All reviews were performed by three lung pathologists (Lee, H., Han, J. and Choi, Y.-L.) and the predominant histological pattern, the presence and proportion of micropapillary patterning, and the necrosis of the primary lung tumors were evaluated.

### 2.3. tDNA EGFR Analysis

We completed tDNA *EGFR* analysis using cobas^®^ *EGFR* mutation test v2 (Roche Molecular Systems Inc., Pleasanton, CA, USA) using FFPE tissue samples from each patient. All tissues were sectioned to 5 μm, deparaffinized, and then subjected to genomic DNA isolation using the cobas^®^ DNA sample preparation kit (Roche Molecular Systems Inc.) according to the manufacturer’s instructions. This genomic DNA was then quantified, and 150 ng of this template DNA was subjected to real-time polymerase chain reaction to amplify the target area and detect the targeted mutations in exon 18, 19, 20, and 21 using a cobas^®^ z480 analyzer (Roche Molecular Systems Inc.). All results were automatically analyzed and collected using cobas^®^ 4800 software (Roche Molecular Systems Inc.). Table S1 summarizes the types of *EGFR* mutations detected using the cobas^®^ *EGFR* mutation test v2.

### 2.4. ctDNA EGFR Analysis

ctDNA analysis was completed using the cobas^®^ *EGFR* mutation test v2 (Roche Molecular Systems Inc.). After collecting whole blood, DNA extraction, RT-PCR, and calculation of a semi-quantitative index (SQI) of mutant DNA were performed according to the manufacturer’s protocol.

### 2.5. Statistical Analysis

All statistical analyses were performed using the IBM SPSS statistical software package (version 24; IBM Corp., Armonk, NY, USA). The correlation between demographic parameters and *EGFR* status was evaluated by Pearson’s chi-square test, paired *t*-test, ANOVA, and logistic regression analysis. The non-normally distributed variables were analyzed by the Shapiro–Wilk test and the Mann–Whitney U test. Overall survival and event-time distribution were analyzed using the Kaplan–Meier method, log-rank test, and Cox proportional hazard model. The *p*-value was considered statistically significant when *p* < 0.05.

## 3. Results

### 3.1. Clinicopathological Characteristics and ctDNA Results

We retrospectively reviewed 554 ctDNA analysis results from 455 patients with advanced lung cancers, either initial or recurrent. Patients who did not undergo tDNA *EGFR* analysis were excluded and 482 cases from 428 patients were left. The demographic data of these 428 patients are summarized in Table 1. The ctDNA *EGFR* analysis was performed after 26.3 months, with the average (range, 0.0–177.1 months; median, 19.3 months) from a diagnosis of NSCLC. A total of 368 patients showed *EGFR* mutation in either ctDNA analysis or tDNA analysis. Among patients with *EGFR*-mutant NSCLC, a total of 236 patients (64.1%) were female and 257 patients (69.8%) were never smokers and were statistically significantly different (*p* < 0.001). The mean age at diagnosis of *EGFR*-mutant NSCLC was 62.65 years (range, 25–91 years; median age, 63 years), which was significantly younger than that of *EGFR*-wild-type NSCLC (mean, 66.40 years; range, 36–83 years; median age, 67 years) (*p* = 0.007). A total of 362 patients (98.4%) with *EGFR*-mutant NSCLC were diagnosed with adenocarcinoma.

**Figure 1:**
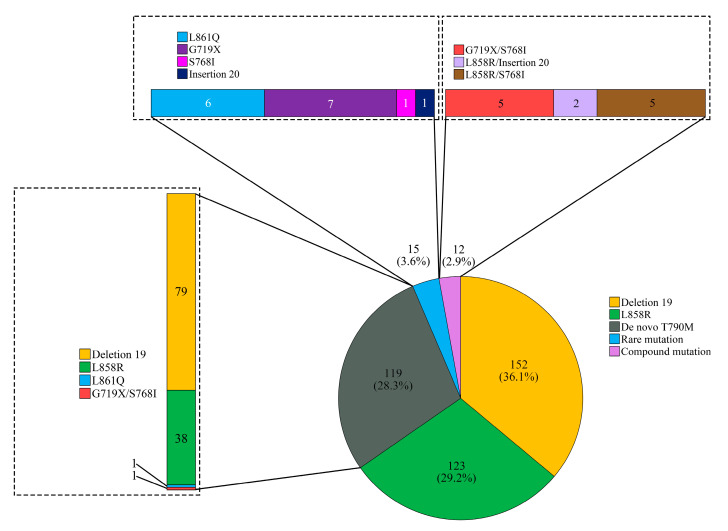
The composition of the *EGFR* mutations. The most common mutation was deletion 19 (152 cases, 36.1%), followed by L858R (123 cases, 29.4%). A total of 119 cases (28.3%) revealed de novo T790M mutation.

To access appropriate results of *EGFR* analysis and compare the effectiveness of ctDNA and tDNA analysis, we excluded 61 *EGFR*-wild-type cases confirmed by both ctDNA and tDNA analysis. After exclusion, 421 cases from 368 patients were finally left. The composition of the *EGFR* mutation type of all 421 cases in accordance with the comprehensive interpretation that was based on the results of both ctDNA and tDNA analyses is visualized in Figure 1. Of the 421 cases, 240 (57.0%) were found to have *EGFR* mutations in the ctDNA analysis. The composition of the *EGFR* mutation type detected by ctDNA analysis is presented in Table 2. It should be noted that there was no significant difference in the detection rate of *EGFR* mutation by ctDNA analysis between the *EGFR* mutation groups (*p* = 0.094).

The clinicopathological characteristics of these patients are summarized in Table 3. The average size of the largest tumor deposit was significantly larger in cases with *EGFR* mutations (ctDNA-positive) than in cases that were *EGFR* mutation-negative (ctDNA-negative) (34.10 mm versus 25.76 mm, *p* < 0.001). A total of 310 cases had progressive disease, defined as an increase in tumor size or number of metastases as confirmed by the radiological review of the CT or PET-CT scans, and 197 (63.5%) cases showed *EGFR* mutation in ctDNA analysis. At the time of ctDNA analysis, metastasis category within the TNM staging protocol was determined based on the M criteria from the American Joint Committee on Cancer staging the 8th edition [26]. When the cases were divided based on M stage, 106, 29, and 286 cases were categorized as M1a, M1b, and M1c, respectively. *EGFR* mutation was detected by ctDNA analysis in 46 (43.4%), 14 (48.3%), and 180 (62.9%) cases of M1a, M1b, and M1c stage, respectively. In addition, 204 cases were subjected to tDNA analysis using tissue obtained from the lung or pleura, and 211 cases underwent tDNA analysis using tissue obtained from various distant metastatic sites, except for the regional lymph node. Among them, 99 (48.5%) cases were detected with *EGFR* mutation in ctDNA analysis, while 138 (65.4%) cases were detected with *EGFR* mutation in ctDNA analysis.

The detection of *EGFR* mutations in the ctDNA assays correlated with advanced metastatic stage at the time of the test and disease progression (*p* < 0.001). In addition, detection of *EGFR* mutations was significantly more common in the ctDNA assays completed in the case where tissue was collected from distant metastatic sites and used to complete a tDNA analysis (*p* < 0.001). When we compared the results of before- and after-treatment samples, the detection of *EGFR* mutations using ctDNA analysis was more frequent in the pre-treatment (treatment naïve) group than in the post-treatment group (75.0% vs. 54.2%) (*p* = 0.003). When we subdivided cases by the history of surgery, the detection rate of *EGFR* mutation of ctDNA analysis was higher in the surgery naïve group than in the post-operation group (60.7% versus 43.2%) (*p* = 0.003).

Since the advanced M stage significantly correlated with the detection rate of *EGFR* mutation in ctDNA analysis, we performed a univariate analysis associated with the type of metastatic organ. The comparison of metastatic organs associated with ctDNA detection is summarized in Table 4.

**Figure 2:**
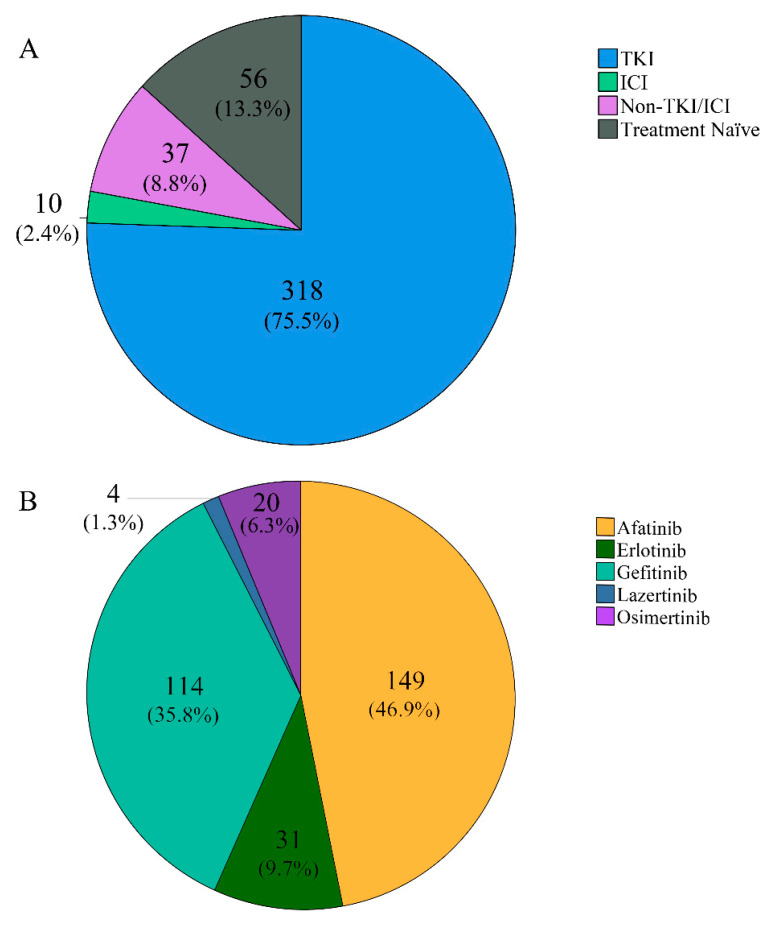
The type of treatment performed in 421 cases (**A**), and the type of tyrosine kinase inhibitor (TKI) administered in 318 cases who underwent TKI treatment (**B**). A total of 318 cases (75.5%) underwent TKI treatment before ctDNA analysis. Among the cases with TKI treatment, the most commonly used regimen was afatinib (149 cases, 46.9%), followed by gefitinib (114 cases, 35.8%), erlotinib (31 cases, 9.7%), osimertinib (20 cases, 6.3%), and lazertinib (4 cases, 1.3%).

Five out of 365 post-treatment cases were not administered TKI, and 42 cases had a change in their treatment plan to something other than TKI. Among 360 cases with a history of TKI administration, the ctDNA *EGFR* analysis was performed on an average of 20.3 months (range, 0.4–80.0 months; median, 17.1 months) after TKI administration. The detailed information of treatment at the time of ctDNA analysis is schematized in Figure 2. A total of 318 cases (75.5%) underwent TKI treatment at the time of the ctDNA analysis, and the most commonly used TKI regimen was afatinib (46.9%).

Multivariate analysis of the clinicopathological parameters affecting the detection of *EGFR* mutations in the ctDNA was completed using the logistic regression analysis model (Table 5). The size of the largest tumor deposits greater than 3 cm was the strongest independent factor associated with the detection rate of ctDNA analysis (odds ratio [OR], 18.216; 95% confidence interval [CI], 2.227–148.983; *p* = 0.007), followed by liver metastasis (OR, 5.684; 95% CI, 1.813–17.820; *p* = 0.003). Additionally, progressive disease (OR, 3.746; 95% CI, 2.213–6.341; *p* < 0.001), an M stage above M1a at the time of ctDNA analysis (OR, 2.015; 95% CI, 1.015–3.999; *p* = 0.045), pleural seeding (OR, 2.088; 95% CI, 1.208–3.607; *p* = 0.008), bone metastasis (OR, 1.968; 95% CI, 1.142–3.393; *p* = 0.015), and a testable amount of tumor tissue obtained from distant metastasis sites (OR, 1.674; 95% CI, 1.049–2.673; *p* = 0.031) were identified as independent factors that significantly increased *EGFR* mutation detection in ctDNA analysis.

A total of 49 patients underwent multiple ctDNA analysis during treatment. Among 49 patients, ctDNA analysis was performed 2 times on 45 patientsand 3 times on 4 patients. After reviewing the disease progression status, 15 patients showed stable or regression of disease after initial ctDNA analysis (stable group), and 34 patients showed progression of disease after initial ctDNA analysis (progression group). The mean period between initial and follow-up ctDNA analysis was 120.24 days (median, 91 days; range, 3–340 days). The mean SQI of follow-up ctDNA analysis was not significantly different between stable group and progression group (*p =* 0.110). The comparison of SQI between initial and follow-up analysis is schematized in Figure S1. In addition, the increment or decrement of SQI was not significantly correlated with overall survival (*p* = 0.226) (Figure S2).

A total of 56 patients with advanced lung cancers underwent ctDNA analysis prior to their first treatment (pre-treatment status) for determining the treatment plan before initial biopsy. The majority of these patients were found to present with detectable *EGFR* mutations in their ctDNA (42/56, 75.0%). About 83.8% (31/37) of patients with M1c disease were detected with *EGFR* mutation by ctDNA analysis, while 53.3% (8/15) of the patients with M1a disease were detected with *EGFR* mutation by ctDNA and was significantly different (*p* = 0.024). Other parameters did not affect the detection rate of *EGFR* mutation in ctDNA analysis in the pre-treatment patients (Table 6). A total of 88 patients underwent resection procedures during the course of their treatment including curative surgery or metastasectomy for tumor removal, with only 38 of these cases (37.5%) shown to be ctDNA-positive. The clinicopathological parameters of patients with a history of surgery are summarized in Table S2. No clinicopathological parameters were shown to be significantly different in these patients. Of the cases in which surgical procedures were performed, a total of 73 underwent histological review of their primary lung cancer or metastatic carcinomas. Micropapillary components, predominant patterns, and the presence of necrosis were all evaluated in this review, but there were no significant differences in the histology of ctDNA-positive and ctDNA-negative cases (Table S3).

### 3.2. Comparison of tDNA and ctDNA Analyses

**Figure 3:**
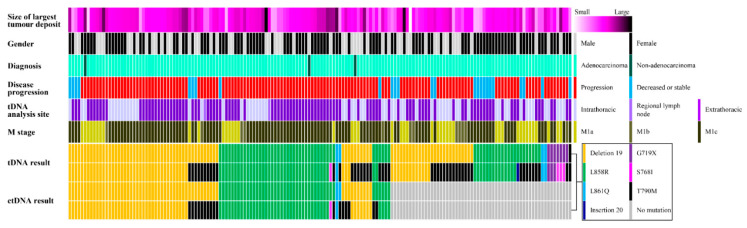
Overview of clinicopathological parameters and the results of the *EGFR* analysis using tDNA and ctDNA. The figure contains clinicopathological information of the disease progression, size of the largest tumor deposits, gender, the site of tumor tissues used for tDNA analysis, metastasis stage, and the results of tDNA and ctDNA analysis. The T790M mutation was detected in 46 cases in tDNA analysis, with only one patient presenting with this mutation alone. For an appropriate comparison of the detection rate of T790M mutation, we excluded 22 cases of ctDNA-negative cases. After exclusion, 28 ctDNA-positive cases were revealed T790M mutation by either tDNA or ctDNA analysis. The number of cases that revealed T790M mutation was 17 (17/28, 60.7%), and 5 (5/17, 29.4%) cases failed to detect T790M mutation in the tDNA analysis.

To accurately compare the detection rate of de novo T790M mutation by ctDNA and tDNA analyses, we selected 164 cases, in which both the ctDNA and tDNA analyses were performed within one month; the results of these assays are schematized in Figure 3. A total of 89 cases (54.3%) exhibited identical results for ctDNA and tDNA analysis, and 59 cases (36.0%) were tDNA-positive and ctDNA-negative.

The clinicopathological parameters associated with the detection of the T790M mutation using ctDNA or tDNA analysis are summarized in Tables S4 and S5. The mean value of the SQI in the non-T790M *EGFR* mutation was the only significant parameter associated with the detection of T790M mutation in ctDNA analysis (mean ± SD, T790M-negative in ctDNA versus T790M-detected in ctDNA; 6.26 ± 4.30 versus 14.44 ± 4.00; *p* < 0.001). The other clinicopathological parameters were not associated with the detection of the T790M mutation by either ctDNA or tDNA analysis.

### 3.3. Relationship between Survival and the Results of the ctDNA Analysis

**Figure 4:**
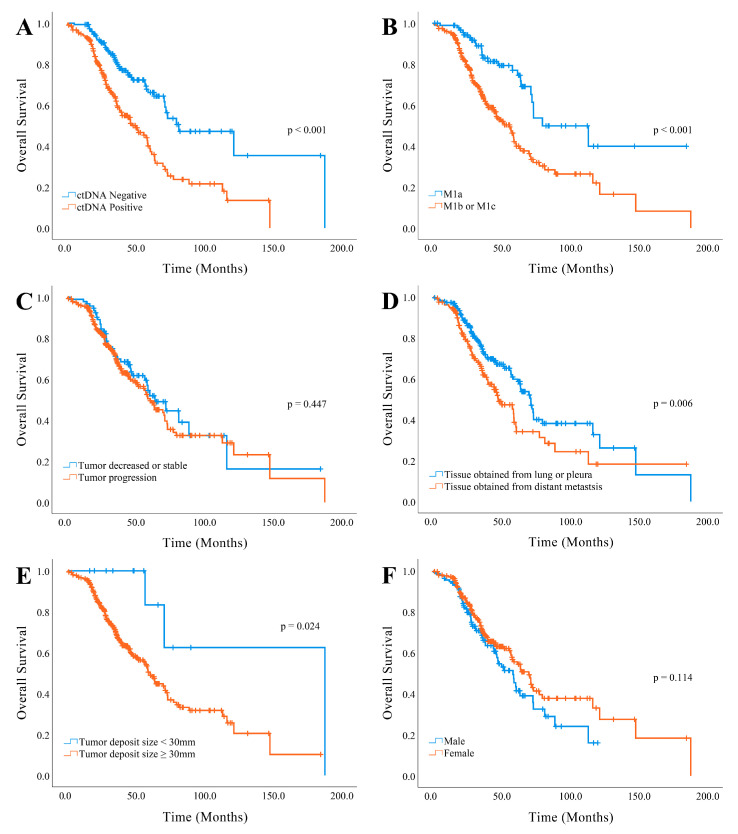
Kaplan–Meier curves for overall survival. The ctDNA status (**A**), advanced M stage above M1a (**B**), the site of tumor tissue obtained for *EGFR* analysis (**D**), and the larger size of the largest tumor deposits more than 30 mm (**E**) were significantly correlated with overall survival. The disease progression (**C**) and sex (**F**) failed to reveal a correlation with overall survival.

The mean follow-up period was 41.09 (range, 0.33–187.44; median, 35.1) months after the initial diagnosis of NSCLC, where 151 patients (41.0%) died during treatment. Overall survival was significantly correlated with ctDNA status (*p* < 0.001), advanced M stage above M1a (*p* < 0.001), the site of tumor tissue obtained for *EGFR* analysis (*p* = 0.006), and the size of the largest tumor deposit 30 mm or above (*p =* 0.024) (Figure 4).

The multivariate analysis was completed using the Cox proportional hazards model (Table 7) and the strongest independent predictor of overall survival was advanced M stage above M1a (hazard ratio (HR), 2.270; 95% CI, 1.461–3.527, *p <* 0.001), followed by ctDNA status (HR, 1.886; 95% CI, 1.319–2.696; *p* = 0.001).

## 4. Discussion

We compared ctDNA analysis and tDNA analysis for the detection of the *EGFR* mutation required for the targeted therapy of NSCLC, and the sensitivity of ctDNA analysis was 57.3%, which was slightly lower than that reported in previous studies [18,19,23,25]. Owing to the relatively high risk of false negatives and inconsistent sensitivity of ctDNA analysis, numerous efforts have been made to reduce these negative outcomes in ctDNA analysis [27,28]. However, there is always a risk of false negatives due to the nature of ctDNA analysis, which relies on the isolation of the circulating cell-free DNA released into the bloodstream from necrotic or apoptotic tumor cells or from circulating tumor cells (CTCs) [24,29,30]. These events can be relatively rare in some cases where tumor burden is low, increasing the risk of false negatives when compared to tDNA assays, which use DNA obtained from the tumor tissue directly. This suggests that there may be room for the complementary application of these analyses to allow for the reduced risk of ctDNA analysis for routine monitoring and the increased sensitivity of tDNA analysis for therapeutic evaluations. It is important to keep in mind that clinicopathological features also interfere with the sensitivity of ctDNA analysis, suggesting that it may be possible to assess which patients are likely to produce false negatives.

Our study revealed that several clinicopathological characteristics are significantly associated with the end results of ctDNA analysis. *EGFR* mutations are more commonly identified in the ctDNA analysis of cases with distant metastasis including liver or bone compared to cases with M1a classification where the cancer is limited to the lung or pleura only. The detection rate of *EGFR* mutation in ctDNA analysis was 62.9% in patients with M1c stage disease, whereas that of patients with M1a stage disease was 43.4%.

The false-negative results of ctDNA analysis can be produced in more than half of the patients with stage M1a even in the stage IV lung cancer. This finding suggests that cases with distant metastasis are likely to have disseminated tumor cells in the blood flow and a higher proportion of ctDNA in the bloodstream, improving the overall sensitivity of the ctDNA analysis. Although the patient is in a metastatic state, the fact that the probability of false negatives is significantly high is once again confirmed in our real-world study as an important point that must be recognized. It is also consistent with our result that *EGFR* mutations are more consistently detected when tDNA evaluations are performed on distant extrapulmonary metastases. The fact that the amount of tissue obtained from metastatic tumors at distant sites is sufficient for tDNA analysis suggests that there is a concomitant increase in the number of CTCs in the bloodstream. Disease progression was also identified as an independent indicator of successful ctDNA analysis, and this suggests that the most significant factor influencing ctDNA sensitivity is the CTCs. On the other hand, it is also noteworthy that the size of the largest tumor deposit is also an independent variable correlated with ctDNA status. This independent correlation, with or without metastasis, whether distant or non-distant, demonstrates that increased release of circulating free DNA from primary tumors is sufficient to improve ctDNA sensitivity.

In addition to the need to know the initial *EGFR* mutational profile of any NSCLC patient, there is a desperate need to facilitate constant monitoring of the genetic profile of patients receiving treatment. In particular, the T790M mutation tends to be acquired during TKI treatment and is known to be responsible for the acquired resistance to TKI [8,9,31] reported in some patients. Identification of this mutation allows clinicians to change their treatment program and apply new treatment agents such as osimertinib [10,32,33,34] to improve therapeutic effect. However, serial biopsy increases post-biopsy risks, including pneumothorax or needle tract seeding. Therefore, ctDNA analysis using plasma or urine could be a feasible alternative for the routine surveillance of patients at risk for *EGFR* mutation.

The *EGFR* mutation detected by ctDNA analysis is more frequent in patients with clinically progressive disease that is refractory to chemotherapy, and ctDNA status can act as an indicator of poor prognosis in patients undergoing treatment. Notably, this provides a basis for faster responses in monitoring or drug changes in ctDNA-positive patients. The lack of correlation between the results of ctDNA analysis in the pre-treatment group also supports the idea that ctDNA analysis is useful as an auxiliary indicator of response to treatment and disease progression.

The role of ctDNA analysis as a screening tool for *EGFR* mutations is a matter of debate. Here, we showed that the ctDNA detection rate for *EGFR* mutations was only 57.3% and that false positives were observed in less than half of these cases but there were a significant number of false negatives. Given that the detection rate increases with advanced or progressive disease, we suggest that this analysis could be useful in estimating the activity of the disease, but that it is insufficient to accurately determine the *EGFR* mutation in patients. The detection rate of *EGFR* mutations in first-time patients increased to 75.0% (42/56), but 25.0% (14/56) of the patients were still negative for *EGFR* mutations when evaluated using a ctDNA analysis. This suggests that if the *EGFR* status is investigated only using ctDNA analysis in the initial patient evaluation, clinicians may miss important *EGFR* mutations impairing their therapeutic efficacy.

In addition, the T790M mutation was not detected by ctDNA analysis in half of the cases with T790M mutation detected by tDNA analysis only. About half of the patients with TKI inhibitor resistance underwent a change in their treatment plan following tDNA analysis, potentially improving their clinical outcomes. Although ctDNA analysis has the huge advantage of being able to evaluate the mutational profile using a non-invasive technique in real time, the risk of false negatives should always be factored into clinical decisions.

By contrast to the low sensitivity of ctDNA analysis, the T790M mutation was found in six tDNA-T790M-negative cases. In other words, the T790M mutation found by ctDNA analysis may not be detected in tDNA due to the heterogeneity of the genetic alterations. Therefore, a comprehensive assessment of *EGFR* mutations using ctDNA and tDNA analysis should be performed on patients throughout treatment to accurately monitor their *EGFR* mutational profile.

We have shown that the complementary application of ctDNA and tDNA analyses is especially important in NSCLC; however, our study does have some potential limitations. First, the follow-up period, especially for the treatment that pertains to naïve patients, was relatively short, thereby suggesting that we may have insufficient data to allow for valid survival and relapse evaluations with these specific patients. In addition, as each of our patients had residual tumors, it was difficult to evaluate relapse-free or progression-free survival rates. Therefore, it is necessary to design an expanded study that includes patients at various stages of lung cancer, as well as those with advanced stage of lung cancer disease.

## 5. Conclusions

Our findings suggest that the detection of *EGFR* mutations in ctDNA samples from patient plasma may be a valuable, minimally invasive method for evaluating lung cancer. In addition, ctDNA-positive patients displayed more progressive and advanced disease characteristics and had worse prognoses compared to ctDNA-negative patients. These findings suggest that ctDNA analysis is an excellent tool for the treatment of patients and the prediction of patient prognosis. However, approximately half of the cases failed to detect *EGFR* mutation in ctDNA analysis and the sensitivity of T790M mutation was slightly more than 50% in ctDNA-positive patients. These findings reveal the relatively low sensitivity of ctDNA analysis in real clinical settings. Therefore, it is important to improve the accuracy of these analyses through the complementary application of both tDNA and ctDNA analyses and to be aware of the risk of false results.

## Figures and Tables

**Table 1:** Demographic parameters of patients.

Variables	*EGFR* Wild Type (*n* = 60)	*EGFR* Mutant (*n* = 368)	*p*-Value
Sex			
Male	37 (61.7)	132 (35.9)	
Female	23 (38.3)	236 (64.1)	<0.001
Age (mean ± SD) (years)	66.40 ± 9.56	62.65 ± 10.753	0.007
Diagnosis			
Adenocarcinoma	50 (83.3)	362 (98.4)	
Transformed small cell carcinoma	0 (0.0)	3 (0.8)	
Non-adenocarcinoma	10 (16.7)	3 (0.8)	<0.001
Smoking status			
Never smoker	27 (45.0)	257 (69.8)	
Ex-smoker	19 (31.7)	77 (21.0)	
Present smoker	14 (23.3)	34 (9.2)	<0.001
Survival status			
Alive	32 (53.3)	217 (59.0)	
Dead	28 (46.7)	151 (41.0)	0.412

Abbreviations: SD, standard deviation. Data are presented as *n* (%) unless otherwise noted. The *p*-value was considered statistically significant when *p* < 0.05.

**Table 2:** The composition of *EGFR* mutation types detected by ctDNA analysis.

*EGFR* Mutation Type	ctDNA-Negative (*n* = 181)	ctDNA-Positive (*n* = 240)	
		Partially Detected * (*n* = 25)	Detected (*n* = 215)
Deletion 19	61 (40.1)	-	91 (59.9)
L858R	62 (50.4)	-	61 (49.6)
Rare mutation	10 (66.7)	-	5 (33.3)
L861Q	3 (50.0)	-	3 (50.0)
G719X	6 (85.7)	-	1 (14.3)
S768I	1 (100.0)	-	0 (0.0)
Insertion 20	0 (0.0)	-	1 (100.0)
De novo T790M mutation	41 (34.5)	22 (18.5)	56 (47.1)
Del19/T790M	27 (34.2)	16 (20.3)	36 (45.5)
L858R/T790M	13 (34.2)	6 (15.8)	19 (50.0)
L861Q/T790M	0 (0.0)	0 (0.0)	1 (100.0)
G719X/S768I/T790M	1 (100.0)	0 (0.0)	0 (0.0)
Compound mutation	7 (58.3)	3 (25.0)	2 (16.7)
G719X/S768I	4 (80.0)	1 (20.0)	0 (0.0)
L858R/Insertion 20	2 (100.0)	0 (0.0)	0 (0.0)
L858R/S768I	1 (20.0)	2 (40.0)	2 (40.0)

Data are presented as *n* (%), unless otherwise noted. * The results of ctDNA analyses found only one of two or more complex mutations defined as partially detected.

**Table 3:** Comparison of clinicopathological parameters between ctDNA-positive and ctDNA-negative.

Variables	ctDNA-Negative (*n* = 181)	ctDNA-Positive (*n* = 240)	*p*-Value
Size of the largest tumor deposits (mean ± SD) (mm)	25.76 ± 15.59	34.10 ± 18.34	<0.001
Diagnosis			
Adenocarcinoma	181 (100)	235 (97.9)	
Non-adenocarcinoma	0 (0)	2 (0.8)	
Transformed small cell carcinoma	0 (0)	3 (1.3)	0.129
History of treatment			
Pre-treatment	14 (25.0)	42 (75.0)	
Post-treatment	167 (45.8)	198 (54.2)	0.003
History of surgery			
Absent	131 (39.3)	202 (60.7)	
Present	50 (56.8)	38 (43.2)	0.003
Disease progression			
Decreased or stable	68 (61.3)	43 (38.7)	
Progression	113 (36.5)	197 (63.5)	<0.001
M stage			
M1a	60 (56.6)	46 (43.4)	
M1b	15 (51.7)	14 (48.3)	
M1c	106 (37.1)	180 (62.9)	<0.001
Site of tissue used for tDNA analysis			
Lung and pleura	105 (51.5)	99 (48.5)	
Lymph node (non-distant)	3 (50.0)	3 (50.0)	
Distant metastasis site	73 (34.6)	138 (65.4)	0.001
Number of cell-free analyses			
1	155 (42.1)	213 (57.9)	
2	24 (49.0)	25 (51.0)	
3	2 (50.0)	2 (50.0)	0.352

Data are presented as *n* (%), unless otherwise noted. The *p*-value was considered statistically significant when *p* < 0.05.

**Table 4:** Comparison of the type of metastatic organ between ctDNA-positive and ctDNA-negative.

Variables	ctDNA-Negative (*n* = 181)	ctDNA-Positive (*n* = 240)	*p*-Value
Number of metastatic organ types * (mean ± SD (median))	2.17 ± 0.95 (2.00)	2.53 ± 1.01 (3.00)	<0.001
Lung-to-lung metastasis			
Absent	66 (36.5)	105 (43.8)	
Present	115 (63.5)	135 (56.2)	0.132
Pleural seeding			
Absent	128 (70.7)	147 (61.2)	
Present	53 (29.3)	93 (38.8)	0.043
Distant lymph node metastasis			
Absent	148 (81.8)	175 (72.9)	
Present	33 (18.2)	65 (27.1)	0.033
Brain metastasis or leptomeningeal seeding			
Absent	85 (47.0)	126 (52.5)	
Present	96 (53.0)	114 (47.5)	0.260
Liver metastasis			
Absent	176 (97.2)	207 (86.2)	
Present	5 (2.8)	33 (13.8)	<0.001
Adrenal gland metastasis			
Absent	173 (95.6)	223 (92.9)	
Present	8 (4.4)	17 (7.1)	0.252
Kidney metastasis			
Absent	177 (97.8)	237 (98.8)	
Present	4 (2.2)	3 (1.2)	0.469
Peritoneal seeding			
Absent	180 (99.4)	235 (97.9)	
Present	1 (0.6)	5 (2.1)	0.469
Bone metastasis			
Absent	125 (69.1)	125 (52.1)	
Present	56 (30.9)	115 (47.9)	<0.001
Soft tissue metastasis			
Absent	179 (98.9)	231 (96.3)	
Present	2 (1.1)	9 (3.8)	0.125

Abbreviations: SD, standard deviation. Data are presented as *n* (%), unless otherwise noted. The *p*-value was considered statistically significant when *p* < 0.05. * Non-normally distributed variable.

**Table 5:** Univariate and multivariate analyses of ctDNA and clinicopathological parameters with *EGFR* mutation detection.

Variables	Univariate Analysis		Multivariate Analysis	
	OR (95% CI)	*p*-Value	OR (95% CI)	*p*-Value
The size of the largest tumor deposit > 3 cm	18.494 (2.396–142.729)	<0.001	18.216 (2.227–148.983)	0.007
The number of metastatic organs *	1.452 (1.185–1.780)	<0.001	0.857 (0.602–1.220)	0.392
History of treatment	0.395 (0.209–0.749)	0.003	1.489 (0.729–3.039)	0.274
History of surgery	0.493 (0.306–0.793)	0.003	1.263 (0.733–2.178)	0.400
Progressive disease	2.757 (1.764–4.308)	<0.001	3.746 (2.213–6.341)	<0.001
Advanced M stage (M1b and M1c)	2.144 (1.373–3.346)	0.001	2.015 (1.015–3.999)	0.045
tDNA analysis from distant metastasis	1.706 (1.152–2.527)	0.007	1.674 (1.049–2.673)	0.031
Pleural seeding	1.528 (1.012–2.307)	0.043	2.088 (1.208–3.607)	0.008
Distant lymph node metastasis	1.666 (1.038–2.672)	0.033	1.569 (0.839–2.934)	0.158
Bone metastasis	2.054 (1.371–3.077)	<0.001	1.968 (1.142–3.393)	0.015
Liver metastasis	5.612 (2.145–14.682)	<0.001	5.684 (1.813–17.820)	0.003

Abbreviations: OR, odds ratio; CI, confidence interval. The *p*-value was considered statistically significant when *p* < 0.05. * Continuous variable.

**Table 6:** Clinicopathological parameters of pre-treatment patients associated with ctDNA analysis.

Variables	ctDNA-Negative (*n* = 14)	ctDNA-Positive (*n* = 42)	*p*-Value
Sex	-	-	-
Male	2 (13.3)	13 (86.7)	
Female	12 (29.3)	29 (70.7)	0.307
Size of the largest tumor deposits (mean ± SD; mm)	38.93 ± 29.48	50.26 ± 23.34	0.207
Diagnosis	-	-	-
Adenocarcinoma	14 (100)	41 (97.6)	
Non-adenocarcinoma	0 (0)	1 (2.4)	1.000
M stage	-	-	-
M1a	7 (46.7)	8 (53.3)	
M1b	1 (25.0)	3 (75.0)	
M1c	6 (16.2)	31 (83.8)	0.024
Tumor DNA tissue-acquired site		-	-
Lung and pleura	8 (36.4)	14 (63.6)	
Distant metastasis site	6 (17.6)	28 (82.4)	0.114
Survival status	-	-	-
Alive	10 (71.4)	30 (71.4)	
Dead	4 (28.6)	12 (28.6)	1.000

Data are presented as *n* (%), unless otherwise noted.

**Table 7:** Univariate and multivariate analysis of survival analysis and clinicopathological parameters.

Variables	Univariate Analysis		Multivariate Analysis	
	HR (95% CI)	*p*-Value	HR (95% CI)	*p*-Value
Sex (male versus female)	1.304 (0.938–1.813)	0.114	-	-
ctDNA detection	2.341 (1.652–3.317)	<0.001	1.886 (1.319–2.696)	0.001
Size of the largest tumor deposit > 3 cm	4.389 (1.083–17.784)	0.024	3.608 (0.869–14.982)	0.077
Progressive disease	1.151 (0.801–1.654)	0.447	-	-
Advanced M stage (M1b and M1c)	2.466 (1.601–3.796)	<0.001	2.270 (1.461–3.527)	<0.001
tDNA analysis from distant metastasis	1.562 (1.131–2.157)	0.006	1.258 (0.906–1.746)	0.17

HR, Hazard ratio; CI, confidence interval. The *p*-value was considered statistically significant when *p* < 0.05.

## Data Availability

The data presented in this study are available on request from the corresponding author. The data are not publicly available due to privacy.

## Supplementary Materials

The following are available online at https://www.mdpi.com/article/10.3390/diagnostics11091695/s1. Figure S1: The comparison of the semi-quantitative index (SQI) between progression group (A) and stable group (B); Figure S2. The Kaplan–Meier curves stratified by the change of the semi-quantitative index (SQI); Table S1: The list of EGFR mutations designed to be detected by the cobas^®^ EGFR mutation test v2; Table S2: Comparison of the clinicopathological parameters of patients with a history of surgery between ctDNA-positive and -negative; Table S3: Comparison of histological findings between ctDNA-positive and ctDNA-negative adenocarcinomas; Table S4: Clinicopathological characteristics associated with ctDNA-T790M status in tDNA-T790M-positive patients; Table S5: Clinicopathological characteristics associated with T790M in tDNA analysis in ctDNA-T790M-positive patients.
