# Supplementary figures and images for: Real-World Analysis of the EGFR Mutation Test in Tissue and Plasma Samples from Non-Small Cell Lung Cancer
Source: Diagnostics (Basel). 2021 Sep 17;11(9):1695. doi: 10.3390/diagnostics11091695 (PMC8465683; doi:10.3390/diagnostics11091695)

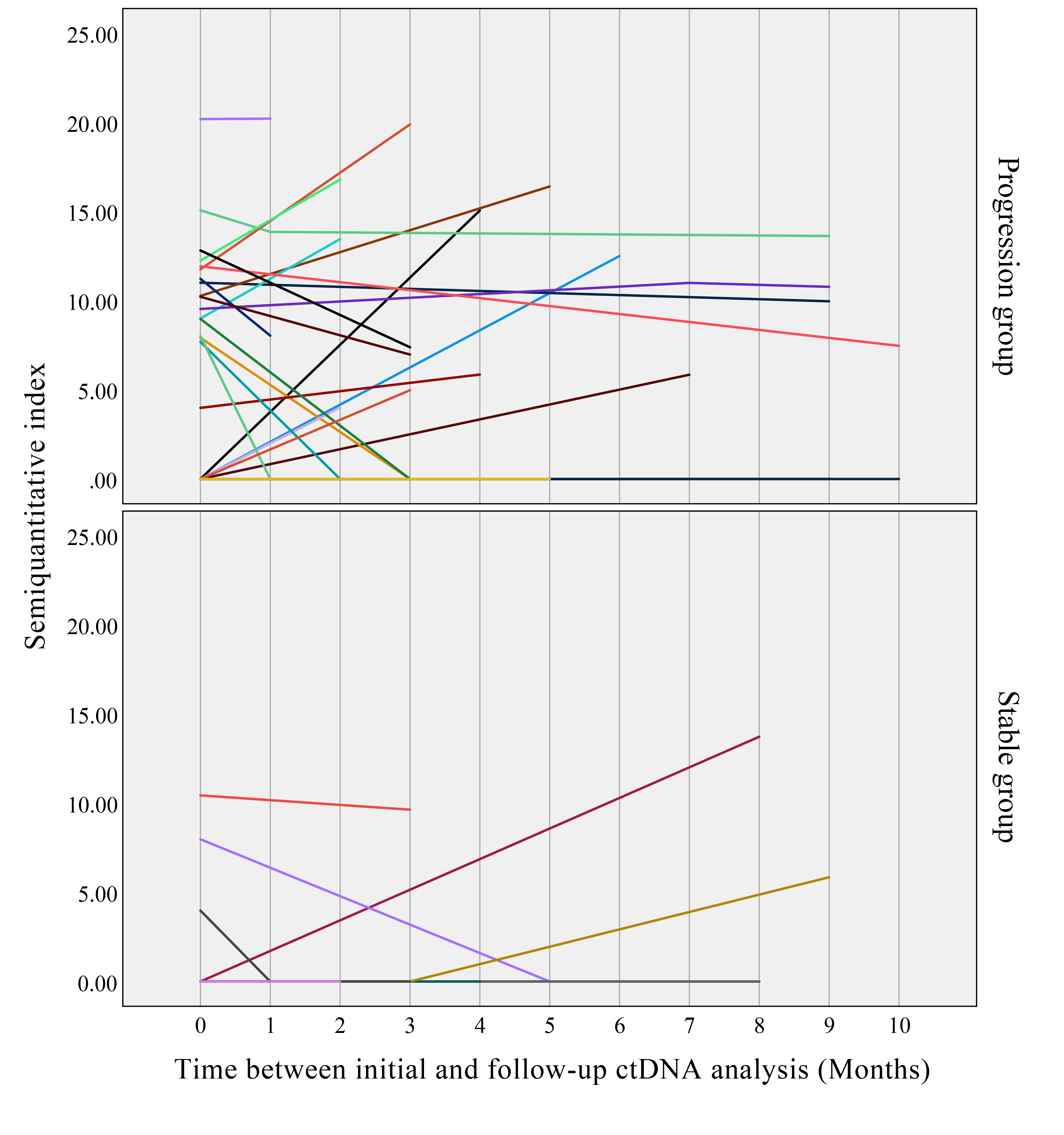

Supplement: Supplementary file 1 [file diagnostics-11-01695-s001.zip › Supplementary Figure S1.tiff]

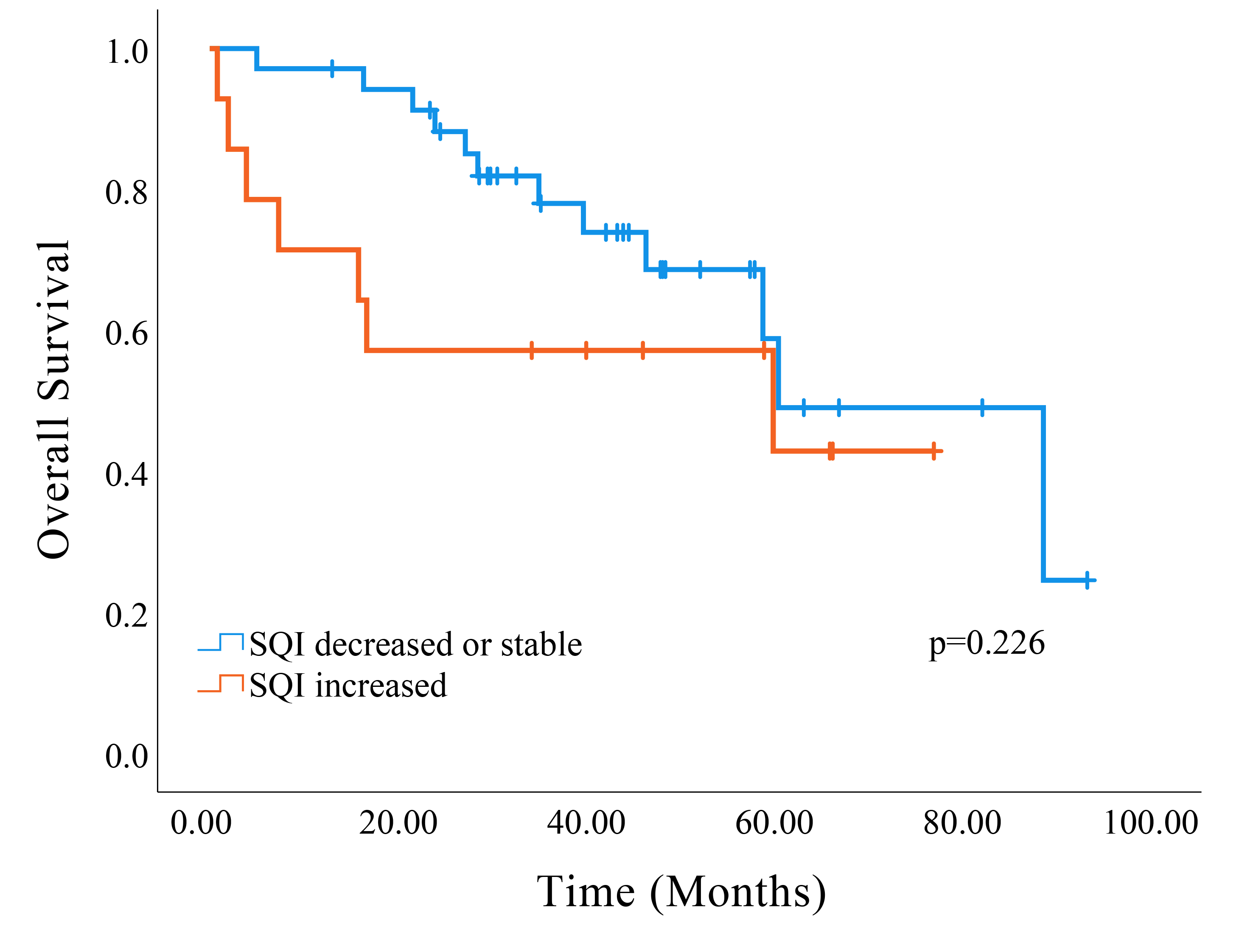

Supplement: Supplementary file 1 [file diagnostics-11-01695-s001.zip › Supplementary Figure S2.tiff]
